# Plantaricin BM-1 enhances anti-colorectal cancer effects by inhibiting CD8+ cytotoxic T cell apoptosis via the ERK/AP1/Bim signaling pathway

**DOI:** 10.3389/fimmu.2026.1792962

**Published:** 2026-03-30

**Authors:** Xuan Zheng, Qi Wang, Xiaodong Song, Jingxin Zhu, Chunyu Dai, Junhua Jin, Congyang Cheng, Hongxing Zhang, Yuanhong Xie

**Affiliations:** 1 Beijing Laboratory of Food Quality and Safety, Beijing Key Laboratory of Agricultural Product Detection and Control of Spoilage Organisms and Pesticide Residue, College of Food Science and Engineering, Beijing University of Agriculture, Beijing, China; 2 Key Laboratory of Dairy Quality Digital Intelligence Monitoring Technology, State Administration for Market Regulation, Inner Mongolia Mengniu Dairy (Group) Co., Ltd., Hohhot, Inner Mongolia, China

**Keywords:** apoptosis, colorectal cancer, cytotoxic CD8+ T cells, plantaricin BM-1, scRNA-seq

## Abstract

**Introduction:**

Colorectal cancer (CRC) remains a leading cause of cancer-related mortality. Plantaricin BM-1, a class IIa bacteriocin from Lactobacillus plantarum, exhibits anticancer potential, but its *in vivo* efficacy against CRC is unclear.

**Methods:**

Using an AOM/DSS-induced CRC mouse model, we administered Plantaricin BM-1 orally and evaluated therapeutic effects via phenotypic, pathological, and inflammatory assessments. scRNA-seq elucidated molecular mechanisms, validated by RT-qPCR, IHC, flow cytometry, and Western blot.

**Discussion:**

ResultsResults demonstrated that Plantaricin BM-1 significantly suppressed tumorigenesis, colon shortening, serum TNF-α levels, and pathological damage. scRNA-seq revealed a 17.38% increase in tumor-infiltrating T cells and a 9.29% expansion of cytotoxic CD8⁺ T cells. Key cytotoxic genes (Gzma, Gzmb, Fasl) were upregulated in CD8⁺ T cells, while the ERK/AP1 pathway was suppressed. Consistently, Plantaricin BM-1 downregulated ERK, AP1, and pro-apoptotic Bim *in vivo* and *in vitro*. Crucially, it inhibited CD8⁺ T cell apoptosis via the ERK/AP1 pathway.

**Discussion:**

These findings provide mechanistic insights for developing Plantaricin BM-1 as an anti-CRC agent.

## 1 Introduction

Colorectal cancer (CRC), which includes both colon and rectal cancers, is the third most commonly diagnosed cancer worldwide and the second leading cause of cancer-related deaths (1). Globally, the incidence of CRC cases is increasing, with more than 1.9 million new cases and over 900,000 deaths reported in 2020 (2). By 2040, the number of CRC cases and CRC-related deaths is projected to reach 3.2 million and 1.6 million, respectively (3). Based on its pathogenesis, CRC can be classified into sporadic, hereditary, and colitis-associated types (4). Colitis-associated colorectal cancer (CAC) develops from inflammatory bowel disease, and its risk progressively increases with inflammatory bowel disease duration, suggesting that persistent inflammation promotes tumorigenesis (5, 6). Although surgical resection, radiotherapy, chemotherapy, and targeted therapy are often used in the clinical treatment of colon cancer, surgical resection and chemotherapy remain the main treatment choices in most countries. The main chemotherapeutic drugs on the market are 5-fluorouracil (5-FU) and oxaliplatin, etc (7, 8). Studies have shown that 5-fluorouracil activates apoptosis by inhibiting DNA synthesis via thymidylate synthase inhibition. In addition, the effect of 5-FU on cell growth arrest and apoptosis has been attributed to the drug’s ability to increase the level and activity of the tumor suppressor p53. However, currently used chemotherapeutic agents often have serious side effects, such as systemic toxicity and acquired resistance, such as mucositis, cardiotoxicity, and bone marrow suppression with 5-fluorouracil (4). Therefore, developing new anti-tumor drugs with high efficacy and fewer adverse effects has become a significant research focus in the biomedical community.

Microorganisms, especially bacteria, can produce proteins and peptides with anti-tumor properties, such as enzymes, toxins, and bacteriocins (9). Bacteriocins, cationic peptides synthesized by ribosomes and secreted by Gram-positive bacteria, have a broad spectrum of antibacterial activity (10). The antimicrobial properties of bacteriocins have long been established, and their anti-CRC activity has gained increasing attention in recent years, particularly due to the serious side effects of chemotherapeutic drugs. Currently, the anti-cancer effects of many bacteriocins have been verified in cellular experiments. For example, the anti-cancer effect of Microcin E492 is mainly manifested through the induction of apoptosis in Jurkat and HeLa cell lines. In contrast, Pyocin S2 inhibits the growth of MCF7 and HepG2 cell lines (9). However, recent research has focused more on animal studies, such as the induction of apoptosis by Nisin ZP via a calpain-dependent pathway in HNSCC cells, with its anti-cancer effect validated *in vivo* using a mouse model of oral cancer (11).

CRC urgently requires the development of an effective drug without side effects due to its high mortality rate. Bacteriocins have become a research focus because they selectively target cancer cells without harming healthy cells. For example, Norouzi et al. treated SW480 CRC cells with different concentrations of Nisin and found that it had significant antiproliferative properties and induced apoptosis through intrinsic pathways, ultimately leading to CRC cell death (12). Previous research on the anti-cancer mechanism of Nigericin in CRC through *in vivo* and *in vitro* experiments revealed that Nigericin directly targeted the β-catenin destruction complex to inhibit the Wnt/β-catenin pathway, playing an anti-cancer role in CRC (13). Plantaricin BM-1 is a novel class IIa bacteriocin produced by the probiotic bacterium *Lactobacillus plantaricin* BM-1 isolated from traditional, naturally fermented meat products. This bacteriocin displays a broad spectrum of inhibition against food spoilage and pathogenic organisms such as *Listeria monocytogenes* (14). Wang et al. demonstrated the anti-cancer ability properties of Plantaricin BM-1for the first time; they found that Plantaricin BM-1 inhibited the proliferation of SW480 CRC cells without affecting normal colonic epithelial cells and induced CRC cell death through a cysteoaspartate-dependent apoptotic pathway following treatment (15). Although extensive research has demonstrated that bacteriocins effectively inhibit tumor cell proliferation and induce apoptosis *in vitro* and in certain *in vivo* models, exhibiting promising anticancer potential, current studies still face significant limitations. Existing findings predominantly focus on exploring *in vitro* mechanisms, while systematic and in-depth investigations into the action mechanisms of bacteriocins within the *in vivo* environment and their regulatory effects on the tumor microenvironment and immune system remain scarce. Against this backdrop, the *in vivo* mechanism of action of Plantaricin BM-1 against colorectal cancer has yet to be fully elucidated, particularly regarding its interaction mechanisms with the tumor microenvironment and immune system, which warrant further exploration.

Apoptosis is essential for maintaining the body’s development and homeostasis in adults. It is a type of programmed cell death that eliminates excess cells, including cancerous, damaged, and immune cells, to maintain balance within the body (16). There is growing awareness that both excessive and insufficient cell numbers can lead to human diseases, such as cancer. However, enhancing the body’s anti-cancer effects can be achieved by increasing the number of immune cells (17–19). Cytotoxic T lymphocytes (CTLs), often referred to as CD8^+^ T cells, express CD8 on their surface and respond to MHC class I-extraneous antigens. They are key components of the adaptive immune system and play an important role in defending against pathogens, such as bacteria, viruses, and tumors. Cytotoxic CD8^+^ T cells are the primary immune surveillance cells that detect antigens in developing malignant cells (20). As major killers of tumor cells, CD8^+^ T cells can eliminate cancer cells through various mechanisms, including the secretion of perforin, granzyme, or membrane Fasl ligands that interact with Fas receptors on cancer cells (21). In the absence of CD8^+^ T cells, the body lacks anti-tumor immunity and becomes less sensitive to tumor growth. Moreover, elevated levels of CD8^+^ T cells in the tumor microenvironment (TME) are associated with active anti-tumor activity in colorectal, cervical, and breast cancers (22). For example, metformin inhibits apoptosis of CD8^+^ T cells and enhances T cell proliferation and cytokine production by decreasing the production of reactive oxygen species, thereby enhancing its anti-tumor effect (23). Consequently, increasing the abundance and activation of CD8^+^ T cells can help inhibit tumor growth and even eliminate tumors (24). Therefore, a better understanding of the pathogenesis of CRC and development of new therapeutic approaches, studying the roles and regulatory mechanisms of CD8^+^ T cells is crucial.

In this study, we investigated the effects of Plantaricin BM-1 on colitis-associated CRC by constructing an azomethane/dextran sulfate sodium (AOM/DSS) mouse model. We found that Plantaricin BM-1 inhibited the apoptosis of cytotoxic CD8^+^ T cells and enhanced its anti-tumor effect through the ERK/AP1/Bim pathway. These findings elucidate how Plantaricin BM-1 improves the CD8^+^ T cell-mediated mechanisms against CRC and provide an experimental basis for further developing Plantaricin BM-1 as an anti-colorectal cancer compound.

## 2 Materials and methods

### 2.1 Preparation of Plantaricin BM-1

*Lactobacillus plantarum* BM-1 was cultured in MRS Medium at 37°C for 12 h and then centrifuged at 10000 rpm at 4°C for 15 min to collect the supernatant. Plantaricin BM-1 was purified using pH-mediated cell adsorption-desorption and cation exchange chromatography, as described in our previous study (14).

### 2.2 Animals and treatment programs

The experimental protocol was approved by the Animal Ethics Committee of the Pony Testing International Group Co., Ltd. (PONY-2023-FL-16). All animal experiments were performed per the guidelines and regulations of the animal experimentation facility. The authors followed the ARRIVE guidelines for all animal experiments. Six-week-old male C57BL/6N mice were purchased from Beijing Vital River Laboratory Animal Technology Co., Ltd. and housed in a pathogen-free environment (22–25 °C, 12 h light/dark cycle) with adequate access to water and food. After a 1-week acclimatization period, the C57BL/6N mice were randomly divided into five groups (n=10 in each group): control, AOM/DSS model, 5-FU, high-dose bacteriocin BM-1 (10240 AU/only/day), and low-dose bacteriocin BM-1 (5120 AU/only/day). Mice received a single intraperitoneal injection of AOM at 10 mg/kg, followed by three cycles of 2% DSS in normal drinking water for 5 consecutive days, followed by normal drinking water for 2 weeks. During the three treatment cycles, mice in the high-dose and low-dose bacteriocin BM-1 groups were gavaged once daily, while mice in the 5-FU group were injected intraperitoneally with 20 mg/kg every 3 days. The mice’s body weights in each group were recorded every 3 days. Finally, the mice were euthanized, spleen tissue was collected, and the length of the colorectum was measured. The colon was incised longitudinally to assess the number of tumors, and the specimens were removed for downstream assays.

### 2.3 Determination of TNF-α and IL-1β concentrations in mouse serum

After collecting blood from the mice, the serum was separated. Cytokines in the mouse serum were then detected using commercially available enzyme-linked immunosorbent assay (ELISA) kits for TNF-α and IL-1β (Nanjing Jiancheng Bioengineering Institute), following the manufacturer’s instructions.

### 2.4 Hematoxylin and eosin staining of CAC tissues from mice

Mouse colorectal tissues were collected and fixed in 4% paraformaldehyde in phosphate buffer for 1 h at room temperature before being embedded in paraffin, as previously described. Tissue sections from paraffin blocks were cut into 4 μm thick slices. Sections were stained with hematoxylin and eosin (H&E) to assess the severity of inflammation, the extent of mucosal damage, and the extent of crypt damage.

### 2.5 Immunohistochemistry

Colorectal tissue paraffin sections were deacetylated with xylene and ethanol, rinsed with water, and soaked in 3% H_2_O_2_ for 10 min thereby removing endogenous catalase. The H_2_O_2_ was poured off and treated with water twice. A citrate buffer solution was added for microwave steaming to expose the antigenic sites. Subsequently, non-specific binding was blocked with 5% bovine serum albumin (BSA) and anti-ERK were incubated overnight at 4 °C. Secondary antibodies conjugated with horseradish peroxidase (HRP) were incubated for 1h at room temperature and stained with DAB color solution and hematoxylin. Microimaging was performed and analyzed using ImageJ.

### 2.6 cDNA library construction and single-cell RNA sequencing

Mouse colorectal tissues were excised and dissociated into single-cell suspensions for quality control and counting, which generally requires ≥ 80% cell viability. The tested cells were washed, resuspended, and adjusted to the appropriate cell concentration (700~1200 cells/μL). The single-cell suspension was loaded onto a 10x Genomics platform, where the cells entered the nanoscale gel column emulsions (GEMs). GEMs contain unique cell barcodes, molecular identifiers (UMIs), primers, enzyme gel beads, and single cells. After the oil-breaking treatment of the GEMs, reverse transcription and amplification were performed. The quality-controlled cDNAs were subjected to second-generation sequencing library construction, followed by quantitative quality control after the fragmentation and ligation of the sequencing junctions. The constructed library was sequenced using a high-throughput sequencing platform in the PE150 sequencing mode.

### 2.7 Process and quality control of the single-cell RNA-seq data

The cells were screened against a range of criteria to retain high-quality cells. Raw data were compared, filtered, barcode-counted, and UMI-counted using Cell Ranger v7.1.0. Utilizing Seurat V4.0 software for further quality control filtering, this paper filtered all cells with less than 201 UMIs, > 6,000 or < 200 expressed genes, a percentage of mitochondrial RNA > 20%, or a percentage of ribosomal RNA < 3% to remove outliers. Finally, 17,233 eligible cells were retained for downstream analysis. For the filtered expression matrix, Seurat uses ‘LogNormalize’ by default, meaning that the expression of each gene is divided by the overall expression of the cell, converted to relative abundance, and then multiplied by a normalization factor (default 10000), followed by a logarithmic transformation. Variable genes were selected based on their average expression and dispersion, with the first 2,000 highly variable genes per sample used by default. Principal component analysis (PCA) was performed using these variable genes. PCA and t-distributed stochastic neighbor embedding (t-SNE) were used to reduce the dimensionality of the first 30 principal components. After downscaling, clustering was performed using Seurat’s default’ SNN’ method with a resolution of 0.8. The underlying principle is that similar cells exhibit similar gene expression patterns, allowing cells of the same type to be clustered based on their gene expression profiles to identify different cell subtypes. The batch effect between samples was removed by default, using a combination of CCA and MNN. Identified clusters and subclusters were visualized using Unified Manifold Approximation and Projection (UMAP) or t-SNE analysis.

### 2.8 Annotating cell clusters

After clustering analysis, the cells were divided into clusters, and comparative analyses were performed between one subpopulation and the remaining subpopulations to identify differential genes. Seurat calculates these differences using the FindAllMarkers function, which uses a differential expression analysis algorithm to identify differentially expressed genes (DEGs) in each cluster, providing a basis for further screening marker genes. The screening thresholds for determining significant differences in gene expression between clusters are set at default values of Padjust < 0.05 and |log2FC| ≥ 0.25. Cell clusters were identified and annotated by matching the specific genes of each cluster with known characteristic genes from the literature and the CellMaker database.

### 2.9 Differential gene expression in the model and high-dose Plantaricin BM-1 groups

Seurat software was used to analyze the differential expression of single-cell transcriptomes, specifically employing the FindAllMarkers function. DEGs were identified based on the following criteria: |log2FC| ≥ 0.25 and Padjust ≤ 0.05. This module allows for comparing differential genes between different samples or groups and within the same cell type for subsequent analyses.

### 2.10 Functional pathway enrichment analysis and defined cell scoring

Cellular DEGs were enriched and analyzed using Goatools Version 1.4.4 software and the KEGG database to determine the functions and metabolic pathways primarily associated with the gene set. Fisher’s exact test was used to control for false positives, and the P value was adjusted using the BH multiple test correction method by default. The critical threshold for the adjusted P-value was set at p < 0.05. In the single-cell data analysis, a set of custom genes was scored using the AddModuleScore function provided in Seurat, which calculates each gene’s score in each cell. The module score for each sample was determined by averaging the scores of all the cells.

### 2.11 Reverse transcription-qPCR analysis

Total RNA was extracted from tissue cells using a TIANGEN kit according to the manufacturer’s instructions and used as a template for reverse transcription–qPCR (RT-qPCR) analysis with the TIANGEN FastKing One-Step Reverse Transcription Fluorescence Quantification Kit. To quantify the results, a delta/delta cycle threshold (ΔCt = ΔCt (treatment group) - ΔCt (control group)) method was applied to determine changes in gene expression levels. The 2^(-ΔΔCt)^ method was used to calculate the fold change in the model, control, and Plantaricin BM-1 treatment groups. The mRNA expression of *β-actin* was used as an internal control gene in all PCR experiments. The primer sequences are shown in **Table 1**.

**Table 1:** Primers used to perform RT-qRCR.

Gene name	Primer direction	Sequence (5’ to 3’)
*Bcl2l11*	Forward Reverse	CAGAACCGCAAGGTAATCC TTCCTCCTGAGACTGTCGTAT
*Jun*	Forward Reverse	TTCTACGACGATGCCCTCA AAGCGTGTTCTGGCTATGC
*Fos*	Forward Reverse	GGTTTCAACGCCGACTA TCCGCTTGGAGTGTATCTG
*Mapk1*	Forward Reverse	CGTGACCTCAAGCCTTCC GAGCCTGTTCAACTTCAATCC
*β-actin*	Forward Reverse	GTGACGTTGACATCCGTAAAGA GCCGGACTCATCGTACTCC

### 2.12 Mouse spleen CD8^+^ T cell isolation and culture

Dissected mouse spleens were ground and passed through a 70 µm filter to prepare a single cell suspension. The cells were lysed by adding ACK erythrocyte lysis buffer (Solarbio, Beijing, China) for 5 min, centrifuged to discard the supernatant and resuspend the cells, which were then filtered through a 70 µm cell strainer, centrifuged to discard the supernatant and resuspended using a sorting buffer. Mouse splenic CD8^+^ T cells were isolated from splenocytes using magnetic beads according to the instructions of Mouse Splenic CD8^+^ T Cell Sorting Kit (BEAVER, Suzhou, China). Isolated CD8^+^ T cells were expanded and cultured using mouse spleen CD8^+^ T cell-specific expansion medium (IMMOCELL, Xiamen, China), and incubated in a CO_2_ incubator at a temperature of 37 °C, 95% air/5% CO_2_ (HF90; Shanghai Lishen Scientific Equipment Co. Ltd., Shanghai, China).

### 2.13 Flow cytometry analysis of apoptosis

Mouse splenic CD8^+^ T cells were inoculated in six-well plates at a density of 1×10^5^/ml and treated with Plantaricin BM-1 (2560 AU/ml, 5120 AU/ml, 7680 AU/ml, and 10240 AU/ml) for 72 h. Cells were collected, washed twice with PBS, and double-stained using the Annexin V-FITC/PI kit (Solarbio) for double staining. Apoptotic cells were analyzed by flow cytometry (NovoCyte; ACEA Biosciences, San Diego, CA, USA). Flow cytometry results showed that apoptotic cells were mainly composed of early (Annexin V-FITC^+^/PI^-^) and late (Annexin V-FITC^+^/PI^+^) apoptotic cells.

### 2.14 Immunofluorescence assay

Tissue sections were deparaffinized, hydrated, and heated to extract antigen. The sections were then incubated with goat serum for 30 min at room temperature. Subsequently, the sections were incubated with primary antibody (CD8) overnight at 4 °C and with anti-goat/rabbit secondary antibody for 2 h at 37 °C. Images were obtained using confocal microscopy. Antibody information: CD8 (Servicebio, Wuhan, China).

### 2.15 Western blot

To assess the effects of Plantaricin BM-1 on ERK and Bim, we treated complete culture medium (negative control), different concentrations of Plantaricin BM-1 (5120 AU/ml and 10240 AU/ml), along with the ERK inhibitor SCH772984 (MCE, Shanghai, China, 0.5 µM, positive control) to treat mouse spleen CD8^+^ T cells, followed by incubation for 1 hour. Cells were then lysed and proteins were extracted using a protein extraction kit (GenePool, Beijing, China). The protein content was determined using the BCA kit. 20 µg of total protein was separated by 10% sodium dodecyl sulfate-polyacrylamide gel (SDS-PAGE) electrophoresis and later transferred to a 0.45 µm polyvinylidene fluoride (PVDF) membrane. Subsequently, the membrane was closed with 5% BSA dilution, primary antibodies were diluted with 5% BSA, and the membrane was incubated with anti-ERK (Servicebio, Wuhan, China), anti-Bim (Servicebio, Wuhan, China), and anti-β-actin (Servicebio, Wuhan, China) were incubated at 4 °C overnight. The membrane was washed three times with TBST (Servicebio, Wuhan, China), and then goat anti-rabbit IgG-HRP (Servicebio, Wuhan, China) was diluted with a diluent containing 5% BSA and shaken for 60 min at room temperature. The membrane was washed three times with TBST, and the PVDF membrane was immersed in the chromogenic solution for 1 min, and then exposed to color development at room temperature. Gray scale values were analyzed using ImageJ software.

### 2.16 Statistical analysis

Data are expressed as mean ± SD and were analyzed using SPSS (version 22.0; IBM, Armonk, NY, USA). The data were plotted using GraphPad Prism 9.3.1 (GraphPad Software) or Origin 2021 software. Comparisons between multiple groups were analyzed using one-way ANOVA with LSD, Tukey, and Tamhane T2 tests. A p-value of < 0.05 was considered significant.

## 3 Results

### 3.1 Plantaricin BM-1 ameliorates pathological symptoms in AOM/DSS-induced CRC mice

**Figure 1:**
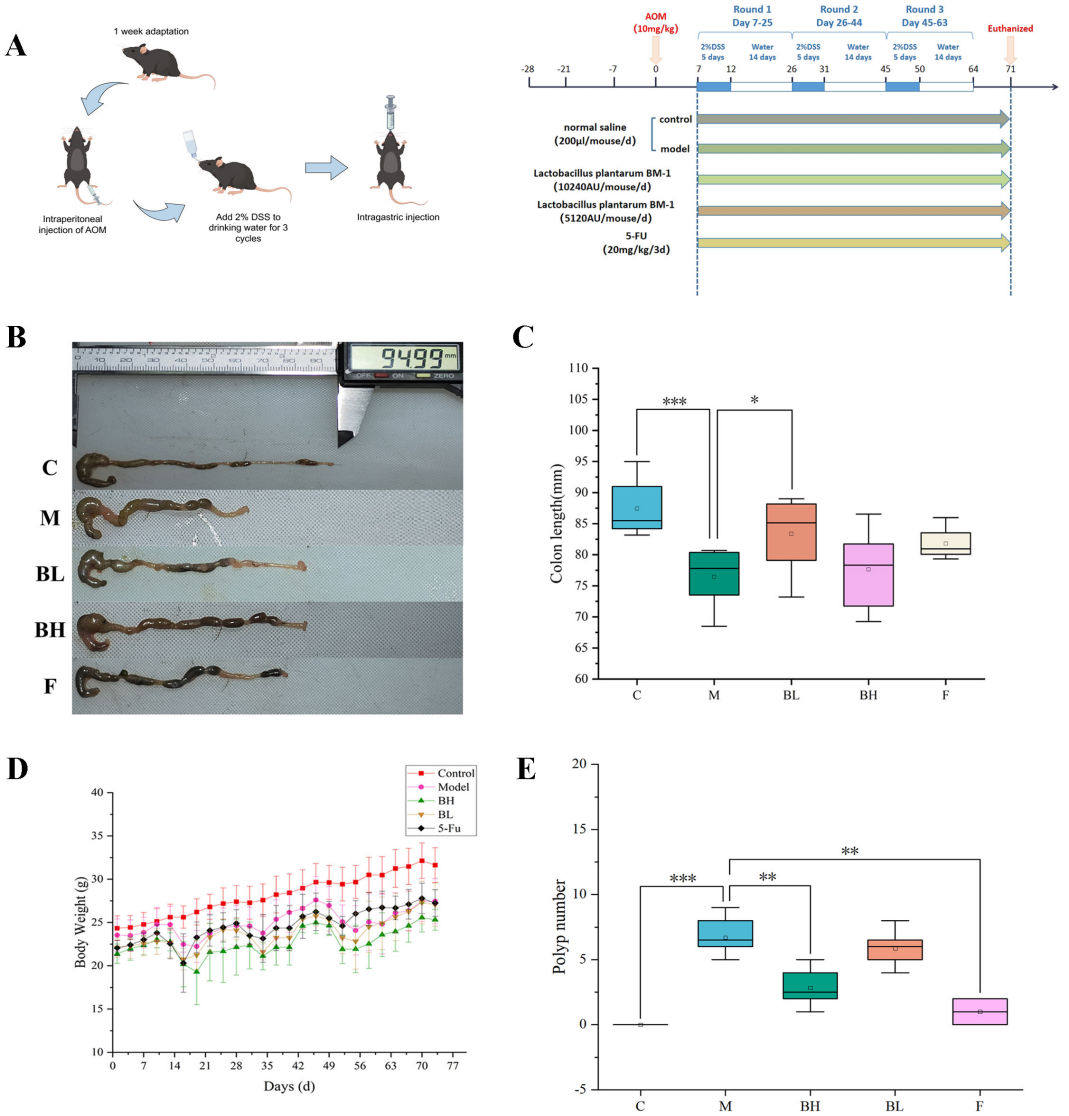
Plantaricin BM-1 ameliorates pathological symptoms in AOM/DSS-induced CRC mice. C, control group; M, model group; BH, high-dose Plantaricin BM-1 group; BL, low-dose Plantaricin BM-1 group; F, 5-fluorouracil group. **(A)** Modeling methods and treatment protocols for AOM/DSS-induced CRC in mice. **(B)** Representative photographs of colorectal length in AOM/DSS-induced CRC mice and normal mice. **(C)** Colorectal length of each group of colitis-associated CRC mice after treatment with different reagents. **(D)** Changes in body weight of mice. **(E)** The number of tumors in each group of colitis-associated CRC mice was treated with different reagents. n = 8. *P < 0.05, **P < 0.01, ***P < 0.001.

In this study, an experimental model of AOM/DSS-induced CRC in mice was established through a single intraperitoneal injection of 10 mg/kg AOM, followed by 2% DSS in drinking water for 5 consecutive days, which was then replaced with normal drinking water for 2 weeks. This cycle was repeated three times. To determine the effect of Plantaricin BM-1 on CRC, mice were administered different doses of Plantaricin BM-1 (high-dose and low-dose) via gavage starting from day one. Equal amounts of saline were administered to the control and AOM/DSS groups (**Figure 1A**). **Figures 1B, C** show that colorectal tissue length was significantly shorter in the AOM/DSS model group than in the control group, and tumors were observed. Compared with the AOM/DSS model group, the 5-FU group showed an increase in colorectal length that did not reach statistical significance (P = 0.509), whereas the Plantaricin BM-1 group exhibited a significant increase. After receiving AOM intraperitoneal injection, the mice gradually lost weight during DSS induction (**Figure 1D**). Throughout the AOM/DSS-induced CRC experiment, mice in the AOM/DSS model group experienced significantly greater weight loss than those in the control group. At the end of the experiment, weight loss remained significantly higher in the AOM/DSS model group than in the control group. Additionally, compared with the AOM/DSS model group, both 5-FU and Plantaricin BM-1 significantly suppressed the number of colorectal tumors. The inhibitory effect of Plantaricin BM-1 on tumor number was dose-dependent, with the highest dose group exhibiting the most pronounced suppression (**Figure 1E**). These findings indicate that Plantaricin BM-1 demonstrated excellent efficacy in improving AOM/DSS-induced CRC symptoms.

### 3.2 Plantaricin BM-1 ameliorates AOM/DSS-induced inflammation and histopathological features in CRC mice

**Figure 2:**
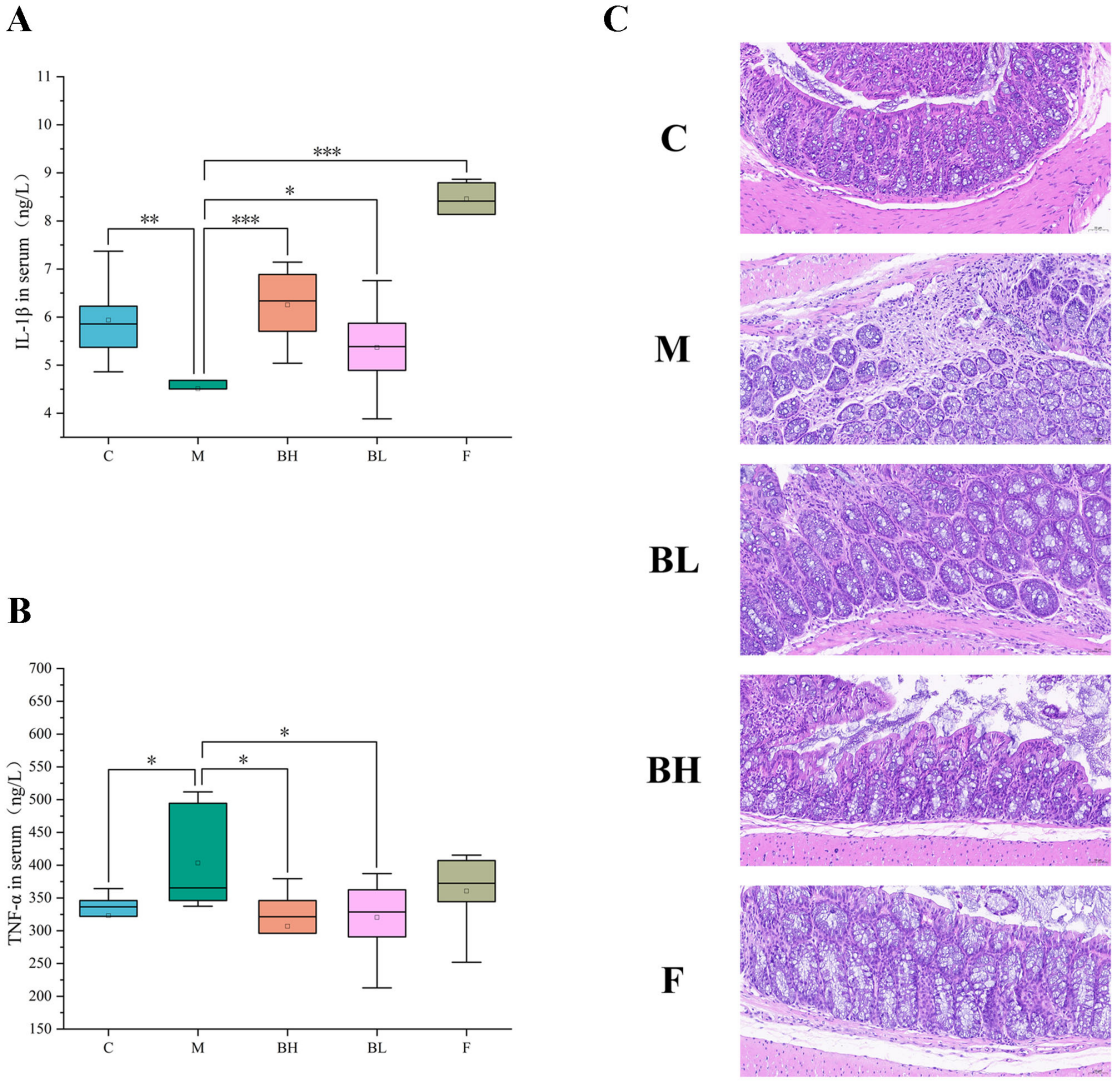
Plantaricin BM-1 ameliorates AOM/DSS-induced inflammation and histopathological features in CRC mice. C: control group; M: model group; BH: high-dose Plantaricin BM-1 group; BL: low-dose Plantaricin BM-1 group; F: 5-fluorouracil group. **(A)** Concentration of IL-1β in mouse serum, measured using an ELISA kit. **(B)** Serum concentrations of TNF-α in mice. **(C)** H&E-stained colorectal pathological sections of normal, AOM/DSS model, Plantaricin BM-1 and 5-FU groups. n = 8. *P < 0.05, **P < 0.01, ***P < 0.001.

To further investigate the effect of Plantaricin BM-1 on AOM/DSS-induced CRC mice, we analyzed changes in the inflammatory factors TNF-α and IL-1β in the serum. The results showed that TNF-α levels were significantly increased in the serum of the AOM/DSS-induced model group compared to the control group. After intervention with Plantaricin BM-1 and 5-FU, TNF-α levels in the serum of CRC mice were significantly reduced compared to the AOM/DSS model group (**Figure 2B**). Additionally, IL-1β levels in the serum were significantly increased in mice treated with Plantaricin BM-1 and 5-FU compared to the AOM/DSS model group, with IL-1β levels in Plantaricin BM-1-treated mice increasing in a dose-dependent manner (**Figure 2A**). Normal colorectal tissue comprises the stroma, submucosa, intestinal epithelium, and mucus layer. CRC is typically accompanied by mucosal ulceration, crypt reduction, decreased cup cells, and other pathological changes (25). **Figure 2C** shows that the control group had structurally normal colorectal tissue with well-ordered glands, whereas the AOM/DSS-induced CRC mice had disorganized colorectal tissue, including absent crypt structure, reduced cup cells, extreme cellular disorganization with abnormal nuclear morphology, and a large number of infiltrating lymphocytes. After treatment with Plantaricin BM-1 and 5-FU, there was a significant increase in cup cells, reappearance of crypt structures, and improvement in abnormal nuclear morphology. These results suggest that Plantaricin BM-1 reduces inflammation and promotes tissue repair.

### 3.3 Plantaricin BM-1 enhances T-cell numbers at the single-cell level

**Figure 3:**
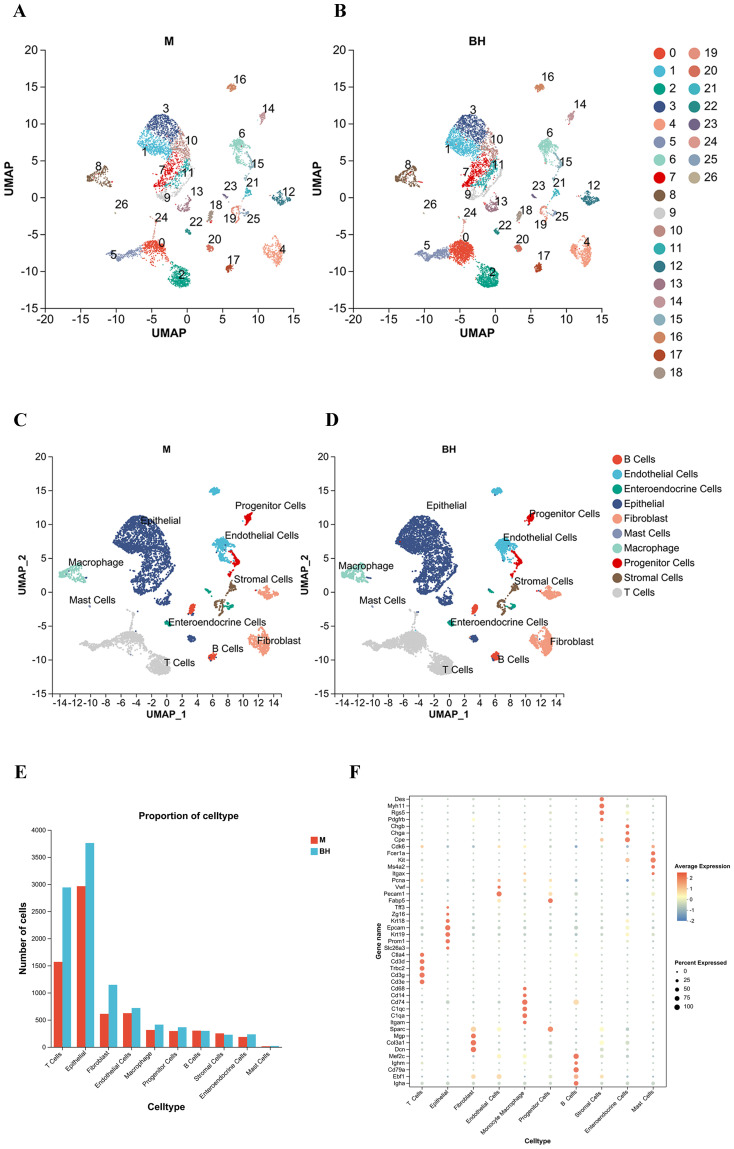
Plantaricin BM-1 enhances T-cell numbers at the single-cell level. **(A)** Cell distribution in the AOM/DSS model group was visualized by UMAP and colored by cluster number in scRNA-seq. **(B)** Cell distribution in the high-dose Plantaricin BM-1 group, visualized by UMAP and colored by cluster number in scRNA-seq. **(C)** Cell distribution in the AOM/DSS model group, visualized by UMAP and colored by major cell type compartments in scRNA-seq. **(D)** Cell distribution in the high-dose Plantaricin BM-1 group was visualized by UMAP and colored with major cell-type compartments in scRNA-seq. **(E)** Histogram showing cell type variation across groups. **(F)** Bubble plots showing the expression of cell type markers. n = 3.

To understand the mechanism of action of Plantaricin BM-1 in the treatment of AOM/DSS-induced CRC, colorectal tissues from mice in the AOM/DSS model group and the high-dose Plantaricin BM-1 group were analyzed using scRNA-seq. Single-cell suspensions from colon tissue were converted into sequencing data using 10×Genomics technology. After quality control and filtering, cell and gene libraries were generated for subsequent analyses. A total of 17,233 live cells were obtained from the sequencing data. Quality control, normalization, and batch effect correction were performed by Seurat, and cell clustering was conducted using the ‘SNN’ method. Each cell type was annotated based on the expression of specific cell type markers (**Figure 3F**). **Figures 3C, D** show the analysis of single-cell sequencing data for 10 dominant cell types, including B cells (*Igha*, *Ebf1*, *Cd79a*, *Ighm*, *Mef2c*), T cells (*Ctla4*, *Cd3d*, *Trbc2*, *Cd3g*, *Cd3e*), epithelial cells (*Tff3*, *Zg16*, *Krt18*, *Epcam*, *Krt19*, *Prom1*, *Slc26a3*), endothelial cells *(Pcna*, *Vwf*, *Pecam1*, *Fabp5*), fibroblasts (*Dcn*, *Col3a1*, *Mgp*, *Sparc*), mast cells (*Cdk6*, *Fcer1a*, *Kit*, *Ms4a2*, *Itgax*), monocyte macrophages (*Cd68*, *Cd14*, *Cd74*, *C1qc*, *C1qa*, *Itgam*), progenitor cells (*Fabp5*), stromal cells (*Des*, *Myh11*, *Rgs5*, *Pdgfrb*), and enteroendocrine cells (*Chga*, *Cpe*, *Chgb*). These cells were organized into 27 cell clusters across the M and BH groups (**Figures 3A, B**). Changes in the composition of cell types between the groups were assessed by analyzing the proportion of each cell type in the M and BH groups. The findings showed that Plantaricin BM-1 selectively increased the number of T cells, monocyte macrophages, and fibroblasts, suggesting that Plantaricin BM-1 treatment modulated the TME in AOM/DSS-induced CRC (**Figure 3E**).

### 3.4 Plantaricin BM-1 increases the number of CD8^+^ T cells at the single-cell level

**Figure 4:**
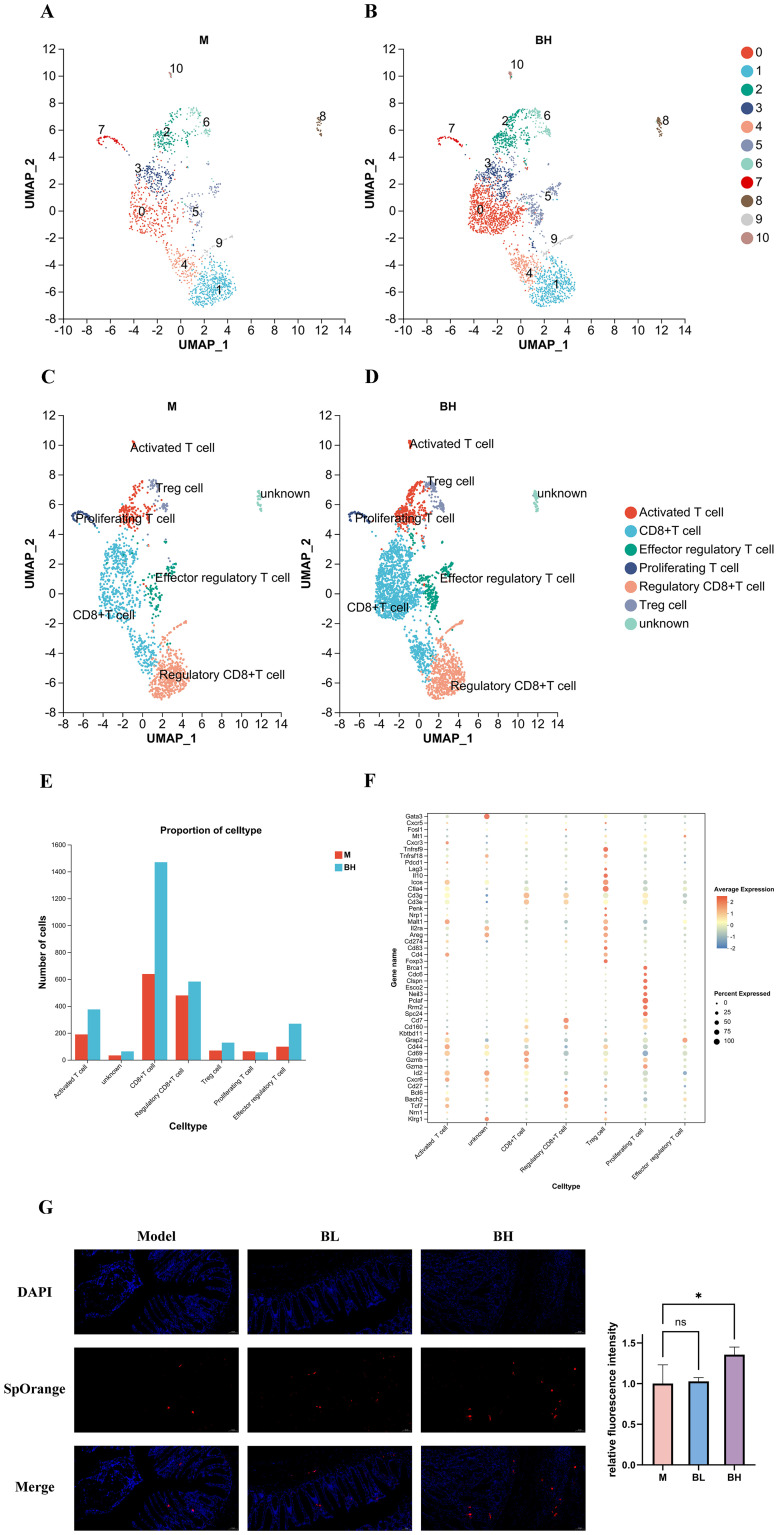
Plantaricin BM-1 increases the number of CD8^+^ T cells at the single-cell level. M: model group; BH: high-dose Plantaricin BM-1 group; BL: low-dose Plantaricin BM-1 group. **(A)** T cell distribution in the AOM/DSS model group, visualized by UMAP and colored by cluster number in scRNA-seq. **(B)** T cell distribution in the high-dose Plantaricin BM-1 group, visualized by UMAP and colored by cluster number in scRNA-seq. **(C)** Distribution of T cell subpopulations in the AOM/DSS model group, visualized by UMAP and colored by major cell type compartments in scRNA-seq. **(D)** Distribution of T cell subpopulations in the high-dose Plantaricin BM-1 group, visualized by UMAP, and colored by major cell type compartments in scRNA-seq. **(E)** Histogram showing changes in T cell subpopulation across groups. **(F)** Bubble plots showing the expression of T cell subpopulation markers. n = 3. **(G)** Immunofluorescence staining showed the expression of CD8, a CD8^+^ T-cell marker, in the colonic tissues of the model group, the high-dose Plantaricin BM-1 group, and the low-dose Plantaricin BM-1 group (n = 8). *P < 0.05, **P < 0.01, ***P < 0.001.

Given the important role of T cells in tumor cells, we further explored the effect of Plantaricin BM-1 on T cells by subjecting those in the BH and M groups to separate cluster analyses, which identified 11 cell clusters (**Figures 4A, B**). Cell clusters were identified and annotated using scRNA-seq, matching markers specific to T cell subpopulations aggregated from the literature and the CellMaker database with genes specific to each cluster. **Figures 4C, D** show that T cells were identified and annotated into six subpopulations: activating T cells (*Cd44*, *Cd69*, *Grap2*), CD8^+^ T cells (*Gzmb*, *Gzma*, *Cxcr6*, *Id2*), effector regulatory T cells (*Cd3g*, *Cd3e*, *Ctla4*, *Icos*, *Il10*, *Lag3*, *Pdcd1*, *Tnfrsf18*, *Tnfrsf9*), regulatory CD8^+^ T cells (*Cd160*, *Cd7*), proliferating T cells (*Brac1*, *Cdc6*, *Clspn*, *Esco2*, *Neil3*, *Pclaf*, *Rrm2*, *Spc24*), and regulatory T cells (*Areg*, *Cd274*, *Cd4*, *Cd83*, *Foxp3*, *Ikzf2*, *Il10*, *Il2ra*, *Malt1*, *Nrp1*, *Penk*). Subsequently, we evaluated the changes in the distribution of T-cell subpopulations among different groups and found that Plantaricin BM-1 increased the number of CD8^+^ T cells compared to the AOM/DSS model group (**Figure 4E**). Next, we further studied the tissue samples of colorectal cancer mice, we performed immunofluorescence CD8^+^ T cell analysis on the samples and observed the number of CD8^+^ T cells under fluorescence microscope using SpOrange staining after treatment with different doses of Plantaricin BM-1. The results showed no visible cells and weak fluorescence intensity in the negative control group. However, the cells in the Plantaricin BM-1-treated group were brighter with stronger fluorescence intensity and the number of CD8^+^ T cells was in a dose-dependent line. In the high-dose Plantaricin BM-1 treated group, the number of cells was significantly higher than that in the low-dose Plantaricin BM-1 treated group (**Figure 4G**). Since CD8^+^ T cells are crucial for anti-tumor immunity, we studied the effect of Plantaricin BM-1 treatment on their functional characteristics to investigate the underlying mechanisms of its action against CRC.

### 3.5 Plantaricin BM-1 acts on CD8^+^ T cells through the MAPK pathway in the T cell receptor signaling pathway

**Figure 5:**
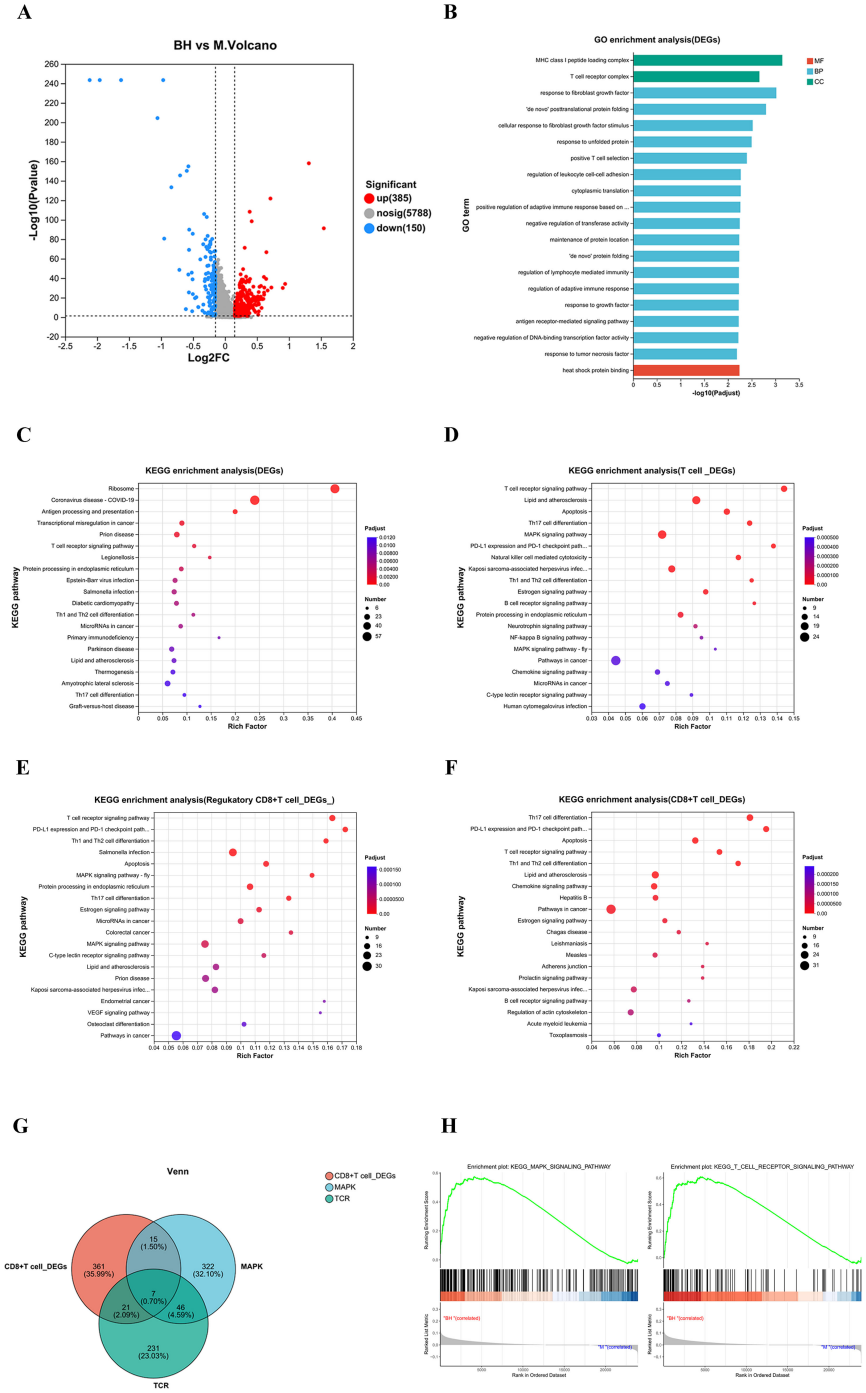
Plantaricin BM-1 targets CD8^+^ T cells via the MAPK pathway within the T cell receptor signaling pathway. **(A)** Volcano plot of DEGs between scRNA-seq samples with or without Plantaricin BM-1 treatment, with red and blue dots indicating significantly up-and down-regulated genes, respectively **(B)** GO enrichment analysis results of DEGs. **(C)** KEGG enrichment analysis results of DEGs. KEGG enrichment analysis results of DEGs in T cells **(D)**, regulatory CD8^+^ T cells **(E)**, and CD8^+^ T cells **(F)**. **(G)** Venn diagram showing overlapping genes among the T cell receptor signaling pathway, MAPK signaling pathway, and DEGs in CD8^+^ T cells. **(H)** GSEA analysis results of the T cell receptor signaling pathway and MAPK signaling pathway in DEGs of CD8^+^ T cells. n = 3.

To study the mechanism of action of Plantaricin BM-1 on CD8^+^ T cells, we elucidated the biological functions and molecular mechanisms of DEGs identified in single-cell sequencing. A total of 535 DEGs were observed in the single-cell sequencing volcano plot, with 385 upregulated and 150 downregulated (**Figure 5A**). Gene Ontology (GO) enrichment analysis of all DEGs revealed two cellular components: the MHCI-like peptide loading complex and the T-cell receptor complex. The main molecular function enriched among the DEGs was heat shock protein binding. Key biological processes included positive T-cell selection, regulation of lymphocyte-mediated immunity, antigen receptor-mediated signaling pathways, and responses to tumor necrosis factor (**Figure 5B**). Additionally, multiple signaling pathways, such as antigen processing and presentation, protein processing in the endoplasmic reticulum, and T cell receptor signaling pathways, were identified through KEGG enrichment analysis of the DEGs (**Figure 5C**). Given that T cells are the most abundant cell type in the TME, we performed KEGG enrichment analysis specifically on DEGs from T cells and CD8^+^ T cells. Notably, we found that the T cell receptor signaling pathway, the MAPK signaling pathway, PD-L1 expression in tumors, and the PD-1 checkpoint pathway were enriched in DEGs from T cells (**Figure 5D**). Moreover, the T cell receptor signaling pathway, PD-L1 expression, and the PD-1 checkpoint pathway were also enriched in regulatory CD8^+^ T cells and CD8^+^ T cells (**Figures 5E, F**). These three signaling pathways play important roles in the growth, development, and death of CD8^+^ T cells. To further investigate the relationship between MAPK signaling pathway and T cell receptor signaling pathway in CD8^+^ T cells after Plantaricin BM-1 treatment, we performed Venn analysis, which revealed that the MAPK signaling pathway gene set, the T cell receptor signaling pathway gene set and the DEGs gene set of CD8^+^ T cells collectively contained seven (**Figure 5G**). Furthermore, gene set enrichment analysis (GSEA) indicated significant enrichment of the T cell receptor and MAPK signaling pathways in CD8^+^ T cells (**Figure 5H**). The Ras-ERK pathway within the TCR signaling pathway is part of the ERK cascade of the MAPK signaling pathway. Collectively, these findings suggest that Plantaricin BM-1 may act on CD8^+^ T cells through the MAPK signaling pathway within the T-cell receptor signaling pathway.

### 3.6 Plantaricin BM-1 inhibits CD8^+^ T cell apoptosis through the ERK/AP1/Bim pathway and enhances CD8^+^ T cell anticancer activity

**Figure 6:**
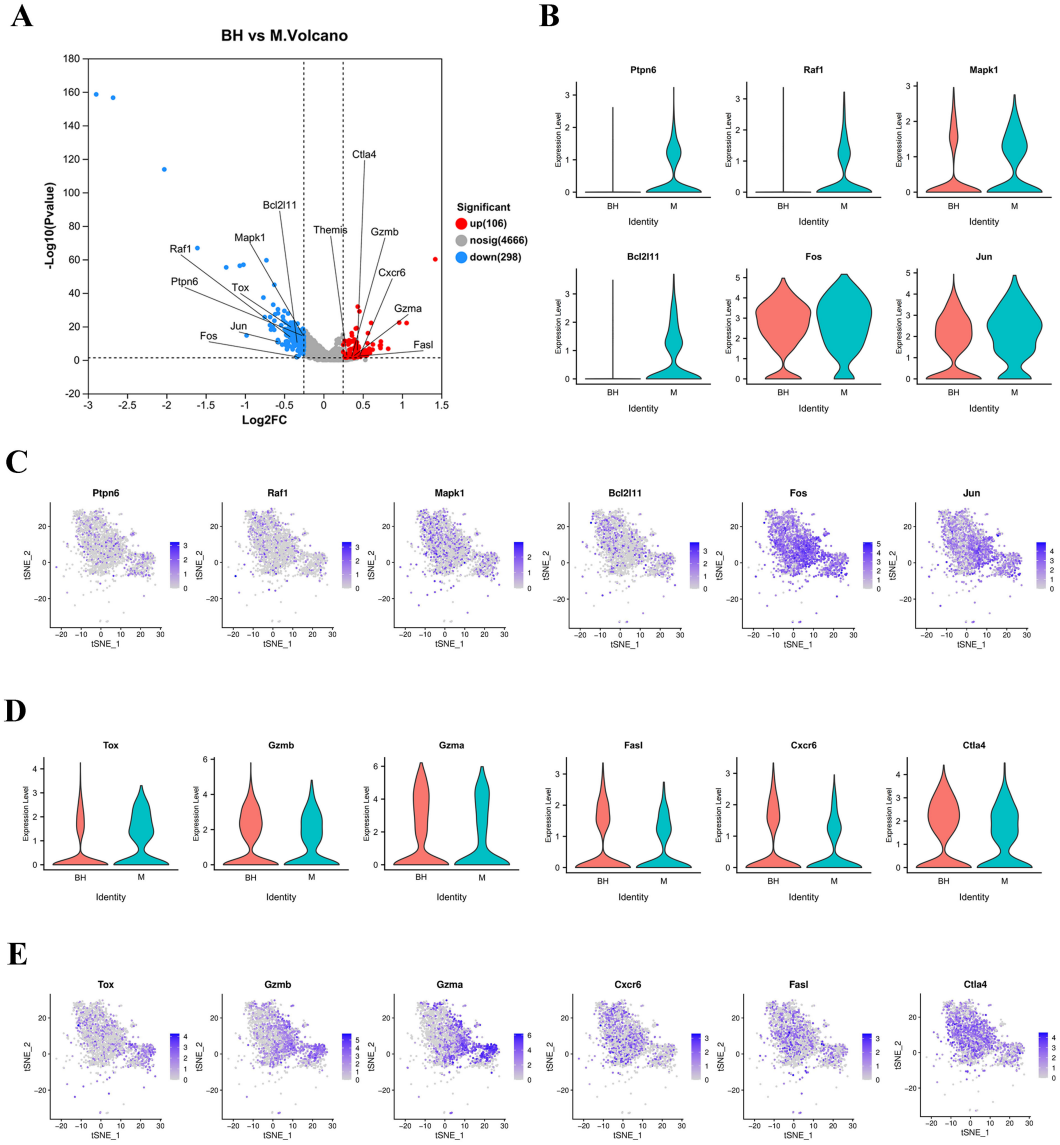
Plantaricin BM-1 inhibits CD8^+^ T apoptosis through the ERK/AP1/Bim pathway and enhances CD8^+^ T anti-cancer activity. **(A)** Volcano plot of DEGs in CD8^+^ T cells between scRNA-seq samples with and without Plantaricin BM-1 treatment. Red and blue dots represent significantly up-and down-regulated genes, respectively. **(B)** Violin plots showing differential expression of *Ptpn6*, *Bcl2l11*, *Fos*, *Jun*, *Mapk1*, and *Raf1* genes in CD8^+^ T cells between scRNA-seq samples with or without Plantaricin BM-1 treatment. **(C)** t-SNE plot showing expression of *Ptpn6*, *Bcl2l11*, *Fos*, *Jun*, *Mapk1*, and *Raf1* genes in CD8^+^ T cells. **(D)** Violin plots showing differential expression of *Gzmb*, *Gzma*, *Fasl*, *Tox*, and *Cxcr6* genes in CD8^+^ T cells between scRNA-seq samples with or without Plantaricin BM-1 treatment. **(E)** t-SNE plot showing the expression of *Gzmb*, *Gzma*, *Fasl*, *Tox*, and *Cxcr6* genes in CD8^+^ T cells. n = 3.

Although preliminary evidence suggests that Plantaricin BM-1 regulates CD8^+^ T cells through the MAPK signaling pathway within the T-cell receptor signaling pathway, the exact mechanisms involved remain unclear. Analysis of the DEGs in CD8^+^ T cells revealed 404 DEGs in the scRNA-seq volcano plot, with 106 upregulated and 298 downregulated genes. *Tox*, *Bcl2l11*, *Fos*, *Jun*, *Mapk1*, *Raf1*, and *Ptpn6* were significantly downregulated, while *Fasl*, *Gzmb*, *Gzma*, and *Cxcr6* were significantly upregulated (**Figure 6A**). This suggests that Plantaricin BM-1 treatment significantly altered gene expression in CD8^+^ T cells. Specifically, Plantaricin BM-1 resulted in a low expression of *Ptpn6*, *Bcl2l11*, *Fos*, *Jun*, *Mapk1*, and *Raf1* in CD8^+^ T cells (**Figures 6B, C**). *Mapk1*, *Raf1*, *Fos*, and *Jun* are associated with the Ras-ERK-AP1 signaling pathway in the TCR pathway of CD8^+^ T cells. Since *Bcl2l11* is a pro-apoptotic gene, Plantaricin BM-1 inhibits apoptosis in CD8^+^ T cells through the ERK/AP1/Bim pathway.

To further investigate the effect of Plantaricin BM-1 on CD8^+^ T cells, we functionally evaluated its cytotoxicity. The expression levels of *Gzmb*, *Gzma*, and *Fasl* were significantly higher in CD8^+^ T cells in the Plantaricin BM-1 treatment group than in the AOM/DSS model group (**Figures 6D, E**) (26, 27). Additionally, reduced *Tox* gene expression was observed in CD8^+^ T cells following Plantaricin BM-1 treatment, a gene known to be associated with T cell depletion (**Figures 6D, E**) (28–30). This suggests that Plantaricin BM-1 treatment inhibits CD8^+^ T cell depletion and prevents a decline in their function. Interestingly, we found that compared to the AOM/DSS model group, Plantaricin BM-1-treated CD8^+^ T cells exhibited high expression of *Cxcr6* (**Figures 6D, E**), which is specifically expressed in CD8^+^ T cells within tumors and plays a role in slowing tumor progression (24). These results demonstrate that Plantaricin BM-1 enhances the cytotoxicity of CD8^+^ T cells and increases the production of cytotoxic molecules in these cells.

Pseudotime analysis revealed the potential of CD8^+^ T cells to transition from an exhausted state toward a functional state: a potential branch indicating reversal from terminal exhaustion to progenitor-like/memory precursor states was observed; exhaustion-related genes (*Ikzf2, Ms4a4b, Layn*) initially increased and then decreased along pseudotime, whereas functional genes (*Kit, Myo1e*) were upregulated at later pseudotime stages, providing a molecular basis for exhaustion-to-function conversion; differences in differentiation trajectories among tumor samples suggest that the tumor microenvironment may regulate this conversion process (**Supplementary Figure S1**).

### 3.7 ERK/AP1/Bim pathway scoring and analysis

**Figure 7:**
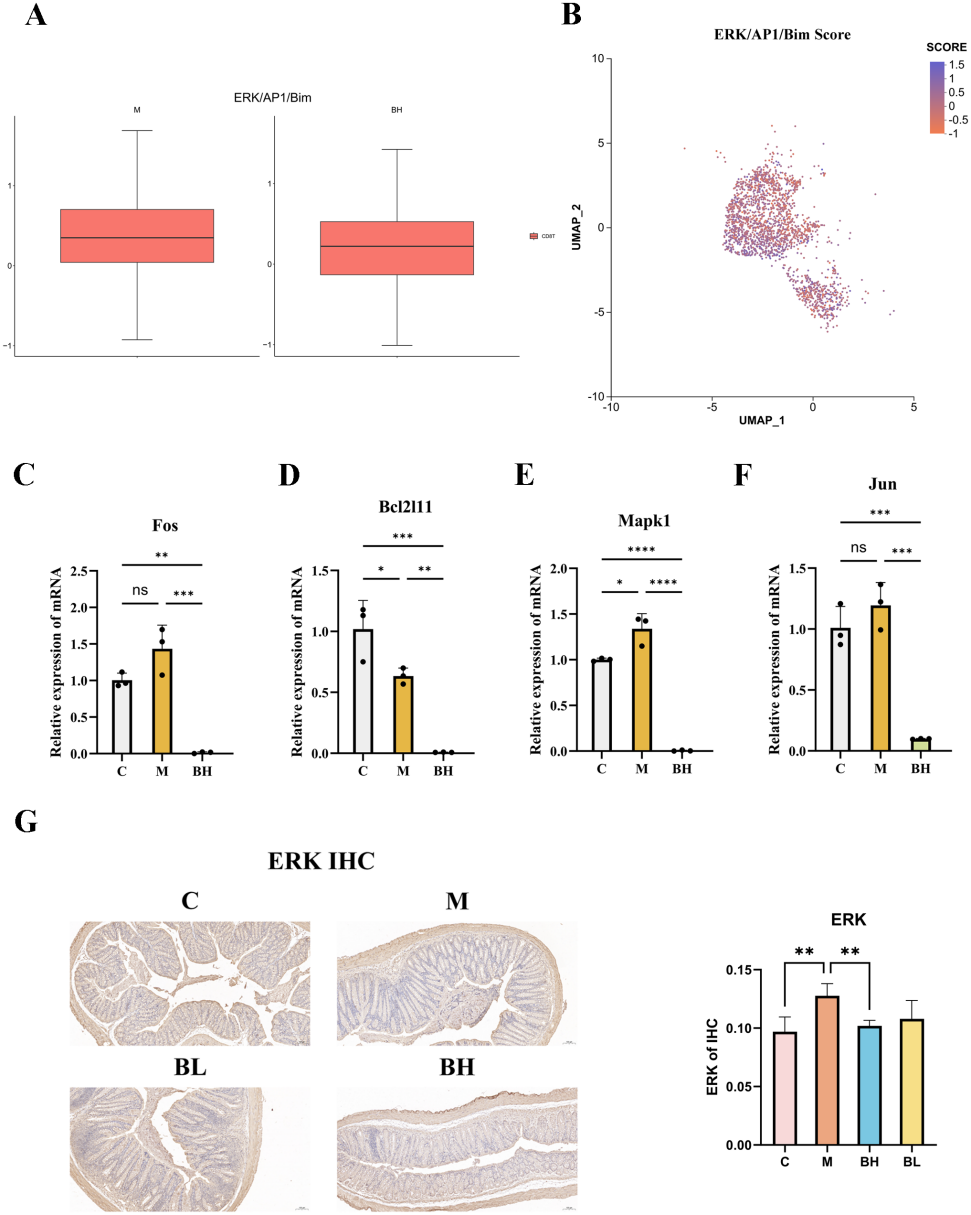
ERK/AP1/Bim pathway scoring and analysis. C: control group; M: model group; BH: high-dose Plantaricin BM-1 group; BL: low-dose Plantaricin BM-1 group. **(A)** Box line plots showing ERK/AP1/Bim pathway scores of CD8^+^ T cells in the AOM/DSS model group and the Plantaricin BM-1 treatment group (n = 3). **(B)** UMAP plots of ERK/AP1/Bim pathway scores, with each cell’s scores represented by varying shades. RT-qPCR analysis of *Fos* **(C)**, *Bcl2l11* **(D)**, *Mapk1* **(E)**, and *Jun* **(F)** mRNA expression in mouse CRC tissues. **(G)** Immunohistochemical analysis of ERK protein expression in colonic tissues. n = 8. *P < 0.05, **P < 0.01, ***P < 0.001, and ****P < 0.0001.

The ERK/AP1/Bim pathway score for CD8^+^ T cells was determined by identifying the genes associated with this pathway, serving as an indicator of the anti-apoptotic effect of Plantaricin BM-1. The scores are presented using box plots and UMAP plots and the results showed that they were significantly higher in the AOM/DSS model group than in the Plantaricin BM-1 group. This suggests that Plantaricin BM-1 inhibited the ERK/AP1/Bim pathway and, consequently, the pro-apoptotic gene *Bim* in CD8^+^ T cells (**Figures 7A, B**). The scRNA-seq analyses indicated that Plantaricin BM-1’s effect on CD8^+^ T cells was mediated by inhibiting the ERK/AP1/Bim pathway and apoptosis-related genes. Colon tissues were collected for analysis to determine the inhibitory effect of Plantaricin BM-1 on pathway-associated proteins *in vivo*. IHC staining was performed on colon sections. The results showed that different doses of Plantaricin BM-1 inhibited the expression of ERK proteins in the colorectal tissues of mice compared with the model group (**Figure 7G**). To validate these findings, RT-qPCR was used to quantify the mRNA expression of pathway genes, corroborating single-cell sequencing results. The analysis revealed that the expression of ERK/AP1/Bim pathway genes and the pro-apoptotic gene *Bim* was significantly reduced in the colonic tissues of Plantaricin BM-1-treated mice compared to the AOM/DSS model group (**Figures 7C–F**). These results were consistent with those of single-cell sequencing analysis and suggest that Plantaricin BM-1 inhibits CD8^+^ T cell apoptosis by suppressing the ERK/AP1/Bim pathway.

### 3.8 Flow cytometry analysis and pathway Western blot analysis of CD8^+^ T cells

**Figure 8:**
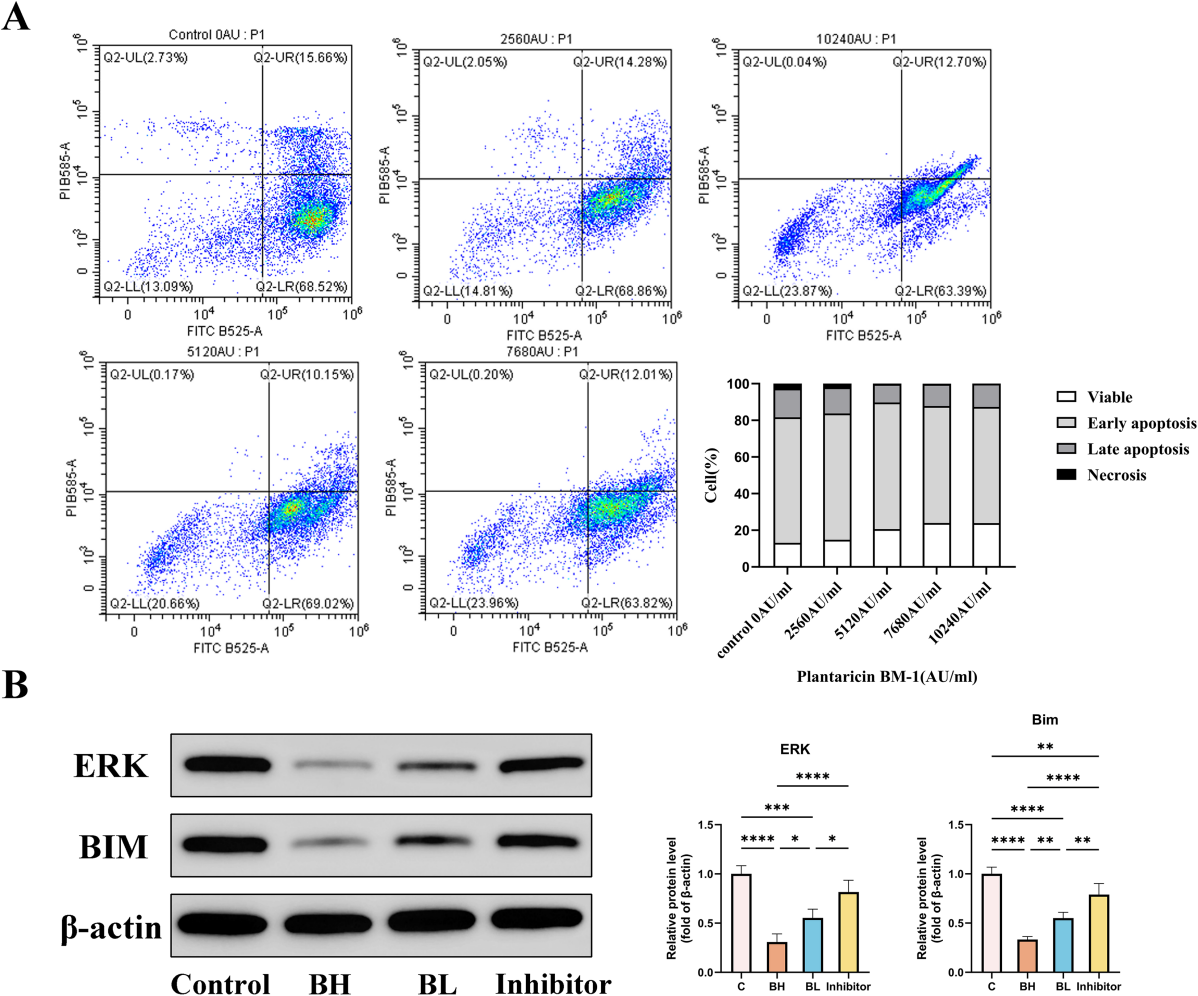
Flow cytometry analysis of CD8^+^ T cells and Western blot analysis of pathway proteins. C: control; BH: high-dose Plantaricin BM-1 (10,240 AU/ml); BL: low-dose Plantaricin BM-1 (5,120 AU/ml); Inhibitor: intervention with ERK inhibitor. **(A)** Flow cytometry detection of apoptosis of mouse spleen CD8^+^ T cells after 72h of the action of different concentrations of Plantaricin BM-1 (blank control, 2560 AU/ml, 5120 AU/ml, 7680 AU/ml, and 10240 AU/ml). **(B)** Western blot analysis of the expression of ERK/AP1/Bim pathway-related proteins (including ERK and Bim) as well as β-actin in the splenic CD8^+^ T cells of mice after treatment with Plantaricin BM-1 (5120 AU/ml and 10240 AU/ml) and inhibitors (n = 8). *P < 0.05, **P < 0.01, ***P < 0.001, and ****P < 0.0001.

We aimed to verify the regulatory effect of Plantaricin BM-1 on ERK/AP1/Bim from the perspective of *in vitro* experiments and to explore whether CD8^+^ T cell apoptosis could be inhibited through this signaling pathway. To achieve this goal, we first investigated the cell survival of CD8^+^ T cells treated with Plantaricin BM-1 for 1 h by flow cytometry using Annexin V-FITC/PI double staining. Under the treatment of 2560 AU/ml, 5120 AU/ml, 7680 AU/ml and 10240 AU/ml of Plantaricin BM-1, the surviving CD8^+^ T cells accounted for 14.81%, 20.66%, 23.96%, and 23.87% of the total cell number, respectively (**Figure 8A**). This experiment showed that Plantaricin BM-1 inhibited CD8^+^ T cell apoptosis in a dose-dependent manner, and the signal detected in the third quadrant was enhanced with increased treatment dose. After treatment with 2560 AU/ml of Plantaricin BM-1, 83.14% of cells remained apoptotic. There was a significant increase in the population of surviving CD8^+^ T cells exposed to 5120 AU/ml Plantaricin BM-1. In CD8^+^ T cells exposed to 7680 AU/ml and 10240 AU/ml of Plantaricin BM-1, cell viability increased and the apoptosis rate decreased significantly. Next, we treated mouse spleen CD8^+^ T cells with different doses of Plantaricin BM-1 and ERK inhibitors and detected the pathway related proteins by Western blot assay. Compared with the control group, the ERK inhibitor group displayed significantly inhibited expression of ERK and led to the reduction of Bim expression (**Figure 8B**). Similarly, Plantaricin BM-1 treatment significantly inhibited the expression of ERK and led to a corresponding decrease in Bim levels compared to the control group. Taken together, our results suggest that Plantaricin BM-1 exerts an anti-apoptotic effect on CD8^+^ T cells by inhibiting the ERK/AP1/Bim signaling pathway, thereby enhancing cytotoxicity. This provides further evidence for the potential application of Plantaricin BM-1 in the treatment of colorectal cancer.

## 4 Discussion

The natural immune response relies on the interaction between the adaptive and innate immune systems. In the context of anti-cancer immunity, the basic modalities of the immune response are surveillance, detection, and destruction of tumor cells. Both innate and adaptive immune responses depend on leukocytes, including cytotoxic CD8^+^ T cells, which are central to cancer immunotherapy and play a crucial role in killing tumor cells (21). Our findings indicate that Plantaricin BM-1, a novel bacteriocin, enhances the number of cytotoxic CD8^+^ T cells in the TME and inhibits CD8^+^ T cell apoptosis by suppressing the ERK/AP1/Bim signaling pathway.

Plantaricin BM-1 is a novel class IIa bacteriocin secreted by *Lactobacillus plantarum* BM-1. Similar to other class IIa bacteriocins, Plantaricin BM-1 exhibits significant antibacterial activity against a range of Gram-positive and Gram-negative bacteria (14). Additionally, bacteriocins exhibit anti-cancer and other biological activities alongside microbial inhibition. Due to their advantages, including blood insolubility and non-toxicity, bacteriocins’ anti-cancer properties have received increasing attention recently (31–33). Varas et al. used a zebrafish xenograft model to study the anti-cancer activity of microcin (MccE492) *in vivo* (34). However, no studies have been reported on bacteriocins related to CRC. The anti-inflammatory potential of bacteriocins was confirmed by Huang et al., who found that bacteriocin Z alleviated lipopolysaccharide-induced mastitis through inhibition of the ERK1/2 and p38 MAPK signaling pathways, thereby playing an anti-inflammatory role (35). Similarly, Bai et al. demonstrated that DHA significantly suppressed the early inflammatory response and subsequent tumor formation in an AOM/DSS model (36). In line with these findings, our results showed that Plantaricin BM-1 reduced the number of tumors and increased colorectal length, supporting its anti-cancer effect. Furthermore, Plantaricin BM-1 exhibited effective anti-inflammatory effects in an AOM/DSS mouse model, in which elevated levels of inflammatory factors are a key feature of CRC tumor development (37). The pathogenesis of CRC can be inhibited by suppressing the increase in inflammatory factors (38, 39). In our experiments, Plantaricin BM-1 significantly inhibited the expression of the pro-inflammatory factor TNF-α, consistent with previous reports on the effect of bacteriocins on TNF-α (40–42). However, the Plantaricin BM-1-treated mice showed elevated levels of the pro-inflammatory factor IL-1β compared to the AOM/DSS group. Il-1β, a member of the IL-1 family, can be produced and secreted by various cells in tumors, such as immune cells, fibroblasts, and cancer cells (43). Through subsequent research, we found that the increase in serum IL-1β levels following treatment with Plantaricin BM-1 may be attributed to an increase in the number of macrophages and fibroblasts. Recent studies have revealed that IL-1β plays a complex dual role in tumor immunity: in specific contexts, acute or local IL-1β signaling can enhance the recruitment and effector function of CD8^+^ T cells, thereby exerting an anti-tumor immune effect (44). Specifically, Frewert et al. found that direct intratumoral injection of IL-1β significantly recruits CD8^+^ tumor-infiltrating lymphocytes (45), while Eeckhout et al. demonstrated that IL-1β enhances the effector function of anti-tumor CD8^+^ T cells, leading to efficient tumor destruction (46). This suggests that in the immune-activated environment induced by Plantaricin BM-1, IL-1β may recruit CD8^+^ T cells to the inflammatory region, thereby enhancing their infiltration into tumor tissue and ultimately triggering an anti-tumor immune response.

The immune system is a complex, dynamic, and plastic biological system composed of multiple cell types that continuously sense and interact with the local microenvironment to maintain homeostasis in the body (47). The TME is formed through interactions among immune cells, tumor cells, and cancer-associated stromal cells (22). In the context of anti-cancer immunity, tumors are frequently infiltrated by immune cells. The activation of the immune system to attack tumors as an anti-tumor therapy has become a prominent research topic (48). However, the immune cells within tumors are diverse and serve different functions. Therefore, it is essential to accurately transcriptionally characterize the different cell types present in tumors (49). scRNA-seq provides an opportunity for detailed analytical resolution. This technique utilizes a high-throughput sequencing approach to uncover cellular complexities at the single-cell molecular level by examining the single-cell transcriptome. It advances our understanding of the different immune cells in the TME and the immune-regulatory mechanisms that control cancer (47). In this study, 10 cell types were identified using scRNA-seq based on the levels of cell type-specific markers. According to the proportions of different cell types, T cells were identified as the main immune cells inhibiting CRC, with their numbers increasing by 1372 cells (17.38%) after treatment with Plantaricin BM-1.

However, most recent studies have focused on analyzing immune cells (dendritic cells, macrophages, and T cells) in both healthy and diseased tissues (47). For instance, Hu et al. demonstrated that the 5-lipoxygenase inhibitor Zileuton negatively regulates macrophage M2-type polarization via the JAK/STAT pathway, thereby inhibiting invasion, metastasis, and EMT in pancreatic cancer (51). In contrast to Hu et al.’s macrophage-centric approach, our findings revealed that Plantaricin BM-1 treatment induces a more pronounced increase in T cell numbers compared to the AOM/DSS model group. Thus, our study primarily focused on T cells. T-cells maintain immune homeostasis through cellular and humoral immune responses (50). CD4^+^ T cells and CD8^+^ T cells are the most important subpopulations of T cells and tumor growth is mainly controlled by these two cell types. To better understand the mechanism of action of Plantaricin BM-1 against CRC, a detailed analysis in conjunction with the main T cell types is essential. scRNA-seq provides an intuitive understanding of cell profiles. In this study, six T cell subpopulations were identified based on cell-type-specific markers using scRNA-seq. According to the analysis of T cell subpopulation occupancy, the number of cytotoxic CD8^+^ T cells in the AOM/DSS model group was approximately 638, which accounted for a lower total proportion of approximately 40.66% of T cells. In contrast, the number of cytotoxic CD8^+^ T cells in the Plantaricin BM-1 group was approximately 1,469, representing a higher total proportion of approximately 49.95% of T cells. Thus, the increase in cytotoxic CD8^+^ T cells may be a contributing factor to the anti-cancer effects of Plantaricin BM-1 against CRC. To further validate Plantaricin BM-1’s impact on intestinal CD8^+^ T cell abundance, we analyzed drug-treated tissue sections using CD8 immunofluorescence staining and confocal microscopy. Plantaricin BM-1 significantly enhanced CD8^+^ T cell fluorescence intensity in a dose responsive manner, indicating elevated cell numbers in intestinal tissues at higher concentrations. Flow cytometry provided more precise quantification than qualitative or semi-quantitative microscopic observations and confirmed this dose-dependent increase. Notably, this effect parallels reports for established immunomodulators taurine and NaCl, where Ping et al. (52) and Scirgolea et al. (53) documented increased intestinal CD8^+^ T cell counts. These findings suggest Plantaricin BM-1 exerts anticancer effects by expanding intestinal CD8^+^ T cell populations, consequently enhancing antitumor immunity.

Single-cell RNA sequencing (scRNA-seq) further elucidated Plantaricin BM-1’s effects on CD8^+^ T cells, revealing upregulated expression of cytotoxicity related genes (*Gzma, Gzmb, Fasl*) post-treatment. Granzymes (GzmA/GzmB) directly lyse target cells via the cytotoxic granule pathway, whereas FasL induces programmed cell death via the death receptor Fas-mediated apoptotic signaling pathway. Collectively, these results demonstrate Plantaricin BM-1 enhances CD8^+^ T cell cytotoxicity, an effect consistent with functional improvements mediated by butyrate (54) and cycloastragenol (55). In addition, based on scRNA-seq analysis, we gained new insights into the CD8^+^ T cell-associated signaling pathways in the context of Plantaricin BM-1 treatment. Specific cell populations, such as T cells, CD8^+^ T cells, and regulatory CD8^+^ T cells exhibited altered gene expression profiles after Plantaricin BM-1 treatment in our research. After analyzing single-cell transcriptomic data from whole cells and several T cell subpopulations, we found that the therapeutic effect of Plantaricin BM-1 was associated with an increased number of CD8^+^ T cells, which may be related to the inhibition of the ERK/AP1/Bim signaling pathway.

ERK, a serine/threonine protein kinase, serves as a central component of the MAPK signaling pathway, where it mediates the transmission of mitogen signals (56). In eukaryotic cells, the MAPK pathway consists of four well-characterized cascades: the ERK, p38, JNK, and ERK5 pathways (57, 58). Within the MAPK/ERK cascade, ERK acts as a key effector protein. It predominantly resides in the cytoplasm and functions as a pivotal regulator of essential cellular processes, including proliferation, differentiation, apoptosis, and immune responses (59, 60). Artesunate significantly increases ERK phosphorylation in CD8^+^ T cells and CD4^+^ T cells and inhibits mitochondrial pathway-mediated apoptosis in a mouse model of sepsis infected with *Pseudomonas aeruginosa* (61). In T cells, the ERK signaling pathway plays a critical role in regulating activation and survival. It has been demonstrated that UA-induced activation of ERK1/2 promotes the expression of the downstream transcription factor AP-1, thereby enhancing T-cell activity and immune function (62). Activator proteins (AP-1) are intracellular transcriptional activators composed of *Jun* and *Fos* that bind to DNA target sequences as homologous or heterodimer complexes to regulate the expression of target genes (63). The expression of Bim, a pro-apoptotic protein encoded by *Bcl2l11*, can be reduced by inhibiting AP1 (64, 65). Our findings revealed that in an AOM/DSS-induced CRC model, the ERK/AP1/Bim pathway of CD8^+^ T cells of mice treated with Plantaricin BM-1 was inhibited, and the expression of the pro-apoptotic gene *Bcl2l11* was significantly downregulated.

Although multiple lines of evidence support that Plantaricin BM-1 exerts anti-tumor effects by modulating CD8^+^ T cells, several limitations remain. First, we lack *in vitro* co-culture validation of the cytotoxic activity of Plantaricin BM-1-pretreated CD8^+^ T cells against colorectal cancer cells (e.g., MC38/CT26). Second, while Plantaricin BM-1 inhibits CD8^+^ T cell apoptosis via the ERK/AP1/Bim pathway, the mechanism by which CD8^+^ T cells recognize Plantaricin BM-1 remains unclear. We hypothesize that Plantaricin BM-1 binds to an unidentified receptor on CD8^+^ T cells, thereby indirectly suppressing this pathway; as an initial exploration, expression profiles of receptor-related genes are provided in the **Supplementary Data Sheet 1**. Third, CD8^+^ T cell depletion experiments (e.g., using anti-CD8 antibodies) were not performed in this study, and the critical role of these cells in mediating the *in vivo* anti-tumor effects of Plantaricin BM-1 requires further validation. Fourth, given the broad regulatory functions of the ERK pathway in multiple cell types, systemic inhibition may pose off-target risks, potentially affecting other immune cells (such as CD4^+^ T cells and macrophages) or interfering with intestinal epithelial repair, leading to immune imbalance. More importantly, whether Plantaricin BM-1 achieves cell-specific recognition through a distinct receptor remains unknown; lack of selectivity would further increase the likelihood of off-target effects. Future studies should focus on receptor identification and functional assays to elucidate the immune recognition mechanism and signaling specificity of Plantaricin BM-1, thereby comprehensively evaluating its therapeutic safety and efficacy as an anti-tumor agent.

## 5 Conclusion

In summary, we established an AOM/DSS-induced colitis-associated CRC mouse model to demonstrate the anti-cancer effect of Plantaricin BM-1 on CRC. This effect was achieved by inhibiting CD8^+^ T cell apoptosis and thus increasing the number of T cells. Mechanistically, Plantaricin BM-1 inhibits CD8^+^ T cell apoptosis by inhibiting the ERK/AP1/Bim signaling pathway. Additionally, Plantaricin BM-1 enhanced the expression of cytotoxic granules and *Fasl*, increasing CD8^+^ T cell cytotoxicity. These results suggest that Plantaricin BM-1 has the potential to regulate the number and function of CD8^+^ T cells, providing a basis for its development as an anti-CRC compound.

## Data Availability

The datasets presented in this study can be found in online repositories. The names of the repository/repositories and accession number(s) can be found in the article/**Supplementary Material**.
