# Supplementary material for: Plantaricin BM-1 enhances anti-colorectal cancer effects by inhibiting CD8+ cytotoxic T cell apoptosis via the ERK/AP1/Bim signaling pathway
Source: Front Immunol. 2026 Mar 30;17:1792962. doi: 10.3389/fimmu.2026.1792962 (PMC13071030; doi:10.3389/fimmu.2026.1792962)
Supplement: Supplementary file 2 [file Supplementaryfile1.docx]

Supplementary Material

**Plantaricin BM-1 enhances anti-colorectal cancer effects by inhibiting CD8+ cytotoxic T cell apoptosis via the ERK/AP1/Bim signaling pathway**

Xuan Zheng^1†^, Qi Wang^1†^, Xiaodong Song^2^, Jingxin Zhu^1^, Chunyu Dai^1^, Junhua Jin^1^, Congyang Cheng^2^, Hongxing Zhang^1,*^ and Yuanhong Xie^1,*^

***Correspondence:**

Hongxing Zhang; Yuanhong Xie

[hxzhang511@163.com](mailto:hxzhang511@163.com) (H.X. Zhang); [xieyuanh@163.com](mailto:xieyuanh@163.com) (Y.H. Xie)


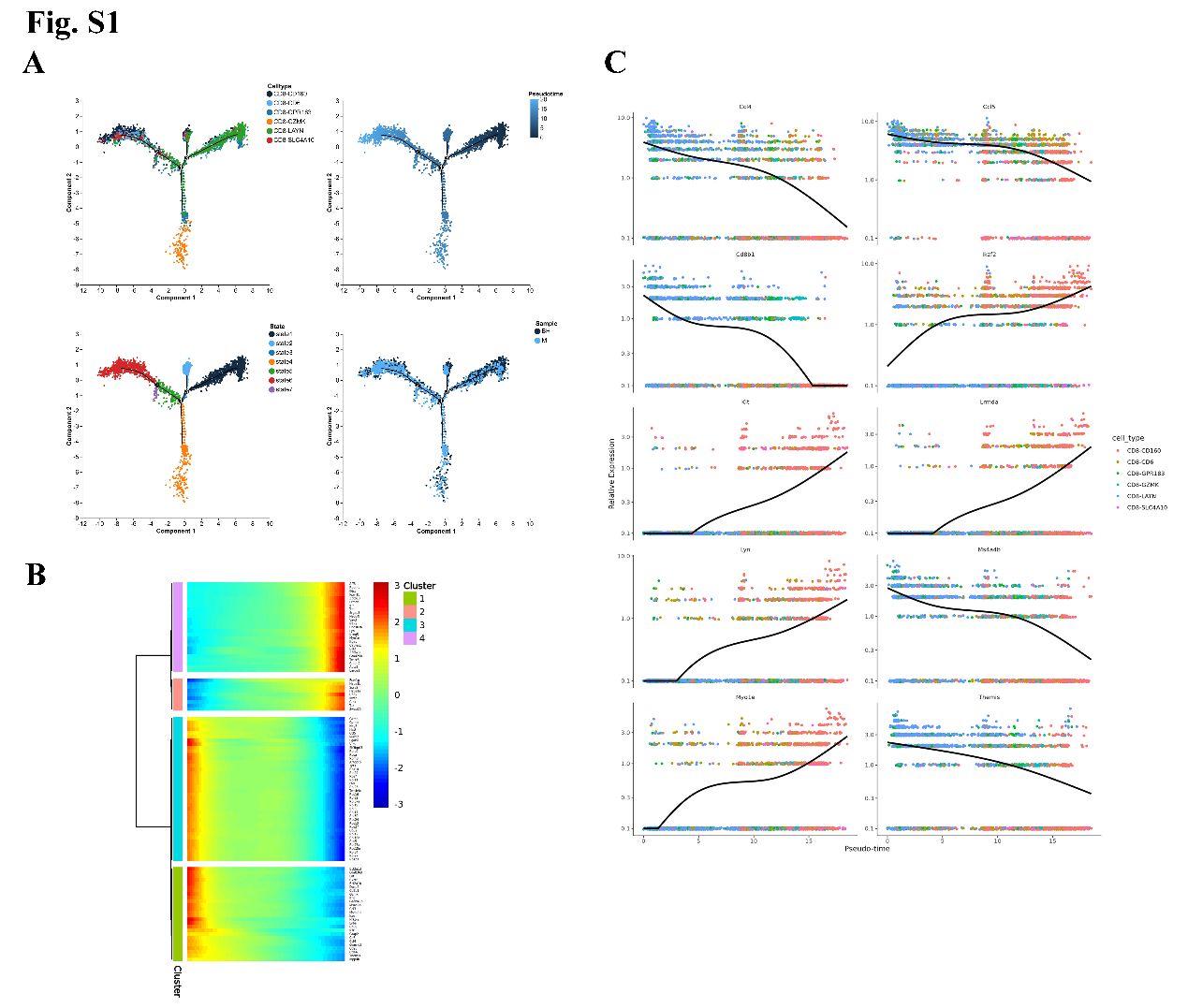


**Figure S1. Pseudotime Trajectory Analysis of CD8⁺ T Cells. Figure 1.** (A) Trajectory distribution of each CD8⁺ T cell cluster over pseudotime across different samples. The right side represents the starting point of development, and the left side represents the endpoint. Cells are color-coded from dark blue (indicating low pseudotime, i.e., early developmental stage) to light blue (indicating high pseudotime, i.e., late developmental stage). (B) Heatmap of pseudotime-related differentially expressed genes. The top 100 most significantly changed genes during differentiation were selected and clustered for visualization. The horizontal axis represents the pseudotime order, and the vertical axis represents different gene cluster modules. Each row corresponds to a gene, and each column represents the average expression value at a given cell state. The color scale ranges from red (high expression) to blue (low expression). (C) Expression dynamics of the top 10 genes along pseudotime.
